# Transcription profiles of non-immortalized breast cancer cell lines

**DOI:** 10.1186/1471-2407-6-99

**Published:** 2006-04-20

**Authors:** Mariana Fernandez-Cobo, James F Holland, Beatriz GT Pogo

**Affiliations:** 1 Department of Medicine, Mount Sinai School of Medicine, New York, New York, USA; 2 Department of Microbiology, Mount Sinai School of Medicine, New York, New York, USA

## Abstract

**Background:**

Searches for differentially expressed genes in tumours have made extensive use of array technology. Most samples have been obtained from tumour biopsies or from established tumour-derived cell lines. Here we compare cultures of non-immortalized breast cancer cells, normal non-immortalized breast cells and immortalized normal and breast cancer cells to identify which elements of a defined set of well-known cancer-related genes are differentially expressed.

**Methods:**

Cultures of cells from pleural effusions or ascitic fluids from breast cancer patients (MSSMs) were used in addition to commercially-available normal breast epithelial cells (HMECs), established breast cancer cell lines (T-est) and established normal breast cells (N-est). The Atlas Human Cancer 1.2 cDNA expression array was employed. The data obtained were analysed using widely-available statistical and clustering software and further validated through real-time PCR.

**Results:**

According to Significance Analysis of Microarray (SAM) and AtlasImage software, 48 genes differed at least 2-fold in adjusted intensities between HMECs and MSSMs (p < 0.01). Some of these genes have already been directly linked with breast cancer, metastasis and malignant progression, whilst others encode receptors linked to signal transduction pathways or are otherwise related to cell proliferation. Fifty genes showed at least a 2.5-fold difference between MSSMs and T-est cells according to AtlasImage, 2-fold according to SAM. Most of these classified as genes related to metabolism and cell communication.

**Conclusion:**

The expression profiles of 1176 genes were determined in finite life-span cultures of metastatic breast cancer cells and of normal breast cells. Significant differences were detected between the finite life-span breast cancer cell cultures and the established breast cancer cell lines. These data suggest caution in extrapolating information from established lines for application to clinical cancer research.

## Background

The search for differentially expressed genes in tumours has made extensive use of array technology. Most studies have involved tumour biopsy samples or established tumour-derived cell lines [1]. Differentially expressed genes may help to identify tumours with high metastatic potential and pathways that might be therapeutic targets.

As noted by Dairkee et al. [2], one drawback of using established cell lines is that the process of immortalization *in vitro *can bias the expression profile when compared to native tissues. Another problem is that a number of widely-used cell lines have sub-lines that differ in their karyotypes and RNA expression levels [3-5]. Biopsies from tumours, unless they are microdissected, contain heterogeneous cell types. A molecular signature (or expression profile) of the whole tumour can be misleading since it is a composite of all the cells, normal and malignant, present in it. Although tumours may consist of several populations of cells that deviate slightly from the clonal strain of origin, metastases may involve only the subline(s) with specific genetic changes that favour metastatic behaviour. Cultures from freshly-derived ascites or pleural effusions may therefore be relatively homogeneous before they acquire the further mutations that immortalize cell lines with unlimited growth potential.

In this communication we report which genes, within a defined set of well-known cancer-related genes, are differentially expressed in freshly-derived breast cancer cell cultures compared with primary normal, established normal and breast cancer cultures. We compared expression patterns in cultures of finite life-span cells from pleural effusions or ascites of breast cancer patients (Mount Sinai School of Medicine: MSSM) with commercially-available finite life-span normal breast epithelial cells (HMECs). To ensure comparability, cultures were used at similar passage numbers and the karyotypes were analyzed. We also compared the expression profiles of some of the most widely-used established breast cancer cell lines and three putatively normal established breast cell lines.

## Methods

### Cultures of finite life-span cells

a): HMECs: Different batches of normal human mammary epithelial cells cultures were obtained (Biowhittaker Molecular Applications Inc., NJ). HMEC specimens were enumerated serially and maintained with MEGM (Clonetics, MD) supplemented with 2.5% FCS (Gibco Invitrogen, CA).

b): MSSMs: Breast cancer cells obtained from ascitic fluids or pleural effusions of patients with breast cancer were seeded and maintained in our laboratory. They were designated MSSM 3 through MSSM 14 and maintained in MEGM supplemented with 2.5% of the corresponding original fluid when available and 2.5% FCS (Gibco Invitrogen, CA). The full characterization of these cultures will be published elsewhere (manuscript in preparation).

HMECs were passaged only twice or thrice in our laboratory. MSSMs were harvested at the 5th or 6th passage after initial plating.

### Cultures of established cell lines

a): Normal-established (N-est.): the cell lines used were derived from normal tissues: MTSV1-7 (a gift from J. Taylor-Papadimitriou), MCF10A (from R Mira y Lopez) and MCF10F (ATCC, MD).

b): Tumour-established (T-est.): cell lines established from breast cancer pleural effusions or tumour tissues: MDA-MB231, MDA-MB453, MDA-MB468, two strains of MCF 7 (designated MCF 7N and MCF 7P), T47D, BT20 and BT474 (all from ATCC, MD). They were maintained in medium and supplements as recommended by ATCC.

All cell cultures were harvested 48–72 h after plating at about 80% confluence. Sources and markers are given in Table 1.

### Arrays

The Atlas Human Cancer 1.2 cDNA expression array (Clontech, CA) is a nylon membrane printed with 200–600 bp fragments of 1176 characterized genes involved in cancer, 9 housekeeping genes and 6 negative controls. RNAs were extracted and labelled with the Atlas pure total RNA labelling system and hybridized according to the manufacturer's instructions.

All the cell lines used for the arrays (9 HMECs, 10 MSSMs, 3 N-est and 7 T-est) were probed twice in separate assays. The accuracy of the duplicates was assessed by Pearson's correlation coefficient based on the adjusted intensities of all the genes spotted on the membrane, which ranged from 0.93 to 0.99.

Hybridizations with 30 μg of total RNA were performed according to the manufacturer's instructions. The hybridized membranes were exposed to a phosphorimager screen and were read at 100 μm resolution in a Storm Phosphorimaging system (Molecular Dynamics, CA). The scanned images were transformed to TIFF files with a PC bit order and then aligned and analyzed using AtlasImage 2.01 software (Clontech, CA). To average or compare the samples, the adjusted intensity signal was normalized using the global normalization mode featured in the software. We report only (a) those genes with significant (p < 0.01) differential expression of 2-fold or more, and (b) genes that were undefined for all the cell lines belonging to one type of sample, but were detected in other types with a difference at least equivalent to one background (540 units in intensity). (Undefined genes are those for which the intensity was below the signal threshold).

The AtlasImage software compares only two samples at a time. When we used it to determine the differences between cell classes, we first averaged the cell lines in the four classes (HMEC, MSSM, N-est and T-est) and then performed the comparisons as instructed by the manufacturer.

Statistical analyses (correlation and two-tailed p values) were performed using Microsoft Excel 2000. Further analyses were performed with Significance Analysis of Microarrays (SAM) [6], Prediction Analysis for Microarrays (PAM) (Stanford Univ., USA), FatiGO [7], Pomelo tool and SVM (Bioinformatics unit, CNIO, Spain [8]) and GoMiner [9].

### Quantitative real-time PCR (Q-PCR)

To validate the results of the cDNA array experiments, some of the genes found to be differentially expressed were further examined by real-time PCR in 10 HMECs, 9 MSSMs, 3 N-est and 8 T-est cell lines. Five μg of total RNA (corresponding to about 100 ng of mRNA) were reverse-transcribed with oligo(dT) (SuperScript II system, Invitrogen, CA) in a 20 μl reaction volume, and after 125-fold dilution, 1.25 μl were used for a 40-cycle PCR on an ABI Prism 7900 thermal cycler. The reaction was carried out in a 384-well plate with a QuantiTect SYBR Green PCR kit (Qiagen Inc, CA) at an annealing temperature of 63°C and detection at 2–5°C below the T_m _of the product as determined from its dissociation curve. Product size was confirmed by agarose gel electrophoresis. The efficiency of each pair of primers for amplification was determined and expression of each gene relative to G3PDH was assessed by the program Qgene [10]. Primers were designed using the program PrimerQuest or Primer3, unless otherwise stated. Primer sequences, lengths and T_m_s of the products are given in Additional file 4.

Samples were tested twice in triplicate. Pearson correlation coefficients for the duplicate Q-PCR results ranged between 0.89 and 0.99.

## Results

### Overall gene expression and class prediction

All the MSSM cultures displayed similar growth rates and had no or minimal chromosomal changes (data not shown). Table 1 shows the main characteristics of these cells.

Each cell line was probed twice. Averages of the duplicates after normalization of the adjusted intensities (as described in the Atlas manual and briefly in Materials and Methods) were used to obtain the expression values for further analysis. The overall gene expression profile, as determined by Pearson's correlation coefficient, discriminates between HMECs and cells derived from metastasis of breast cancer (MSSMs). As seen in Table 2 (and detailed in Additional file 1) the correlation within cell classes was > 0.90; the correlation between cell classes was < 0.82.

Hybridization signals of 928 genes (78.9%) were represented in at least three cell lines. (One class, N-est, comprised three cell lines; the other three classes, HMEC, MSSM and T-est, comprised more.) We used this subset of 928 genes, plus the 9 control genes, to construct a classification model using two prediction programs: PAM and SVM. Since these algorithms do not work well when the numbers of members differ among classes, we trained the SVM program using comparable numbers of lines from three classes (8 HMECs, 9 MSSMs and 7 T-est) and treated the three N-est cell lines and the remaining HMECs and MSSMs as unknowns to be classified. The model thus generated had a classification accuracy of 100% (24/24) by the leave-one-out cross-validation method. The prediction for the three N-est lines was T-est, suggesting that these cells have an expression profile resembling those of the other established cell lines rather than the cultures of finite life-span cells. The remaining HMEC and MSSM cell cultures were correctly assigned to their respective classes (see Additional file 5).

**Figure 1:**
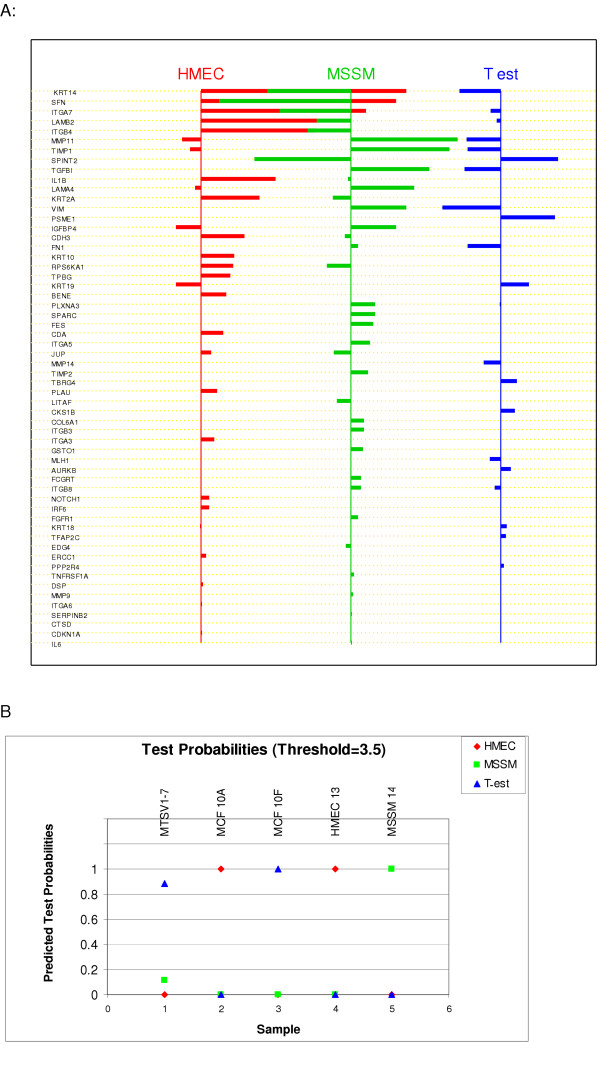
**PAM (Prediction Analysis of Microarrays)**. This is a statistical technique for class prediction from gene expression data using nearest shrunken centroids. This method identifies the subsets of genes that best characterize each class. a) Plot of the shrunkencentroids for the specified threshold with the ranked list of significant genes that are used for the predictions. b) Prediction and probabilities of the test cell lines (MTSV1-7, MCF10A, MCF10F, HMEC 13 and MSSM 14).

**Figure 2:**
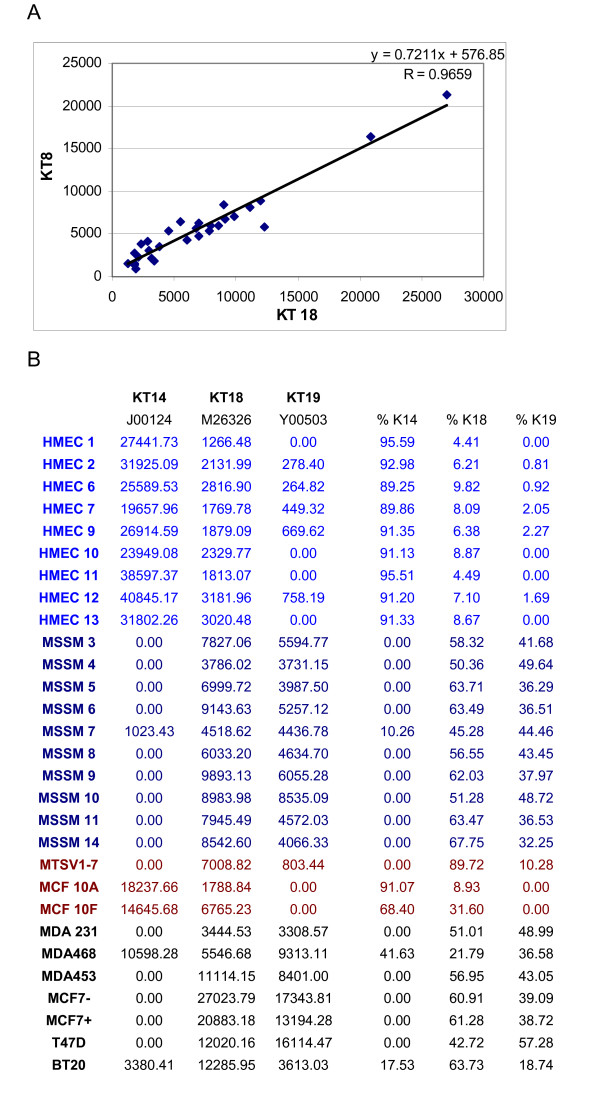
**Expression of KT14, KT18 and KT19**. a) Plot showsthe correlation of the adjusted intensity (AI) signal of the pair KT8/KT18 for each cell line. b) AI of KT14, KT18 and KT19 of each cell line and its relative percentage.

The same analysis with PAM, applying a threshold of 3.5, which gives the maximum number of significant genes yielding no misclassification error upon cross-validation, predicted MTSV1-7 and MCF 10F as members of the "T-est" class and MCF 10A as an HMEC-class member with a probability of 1. Again, HMEC 13 and MSSM 14 were properly assigned (Fig 1 and Additional file 2). Further to elucidate the classification of MCF10A, which is the most widely-used putatively normal breast cell line in array analysis, we examined the expression values of KT14, KT8/18 and KT19 (Fig. 2A and 2B) and CD104. The MCF10A values were similar to those of the HMECs.

### Comparison of non-immortalized normal and cancer breast cells

Of the 1176 genes in the array, 862 (73.3%) gave hybridization signals in at least three of the finite life-span cell lines used. Of these, 123 (14.3%) showed differential expression when HMEC and MSSM cells were analyzed using the AtlasImage software (ratio >2 and difference in adjusted intensity > 540), and 101 were deemed significant genes with SAM (q-value: 0.7298, median #FDR: 0.73718) (Additional file 6).

**Figure 3:**
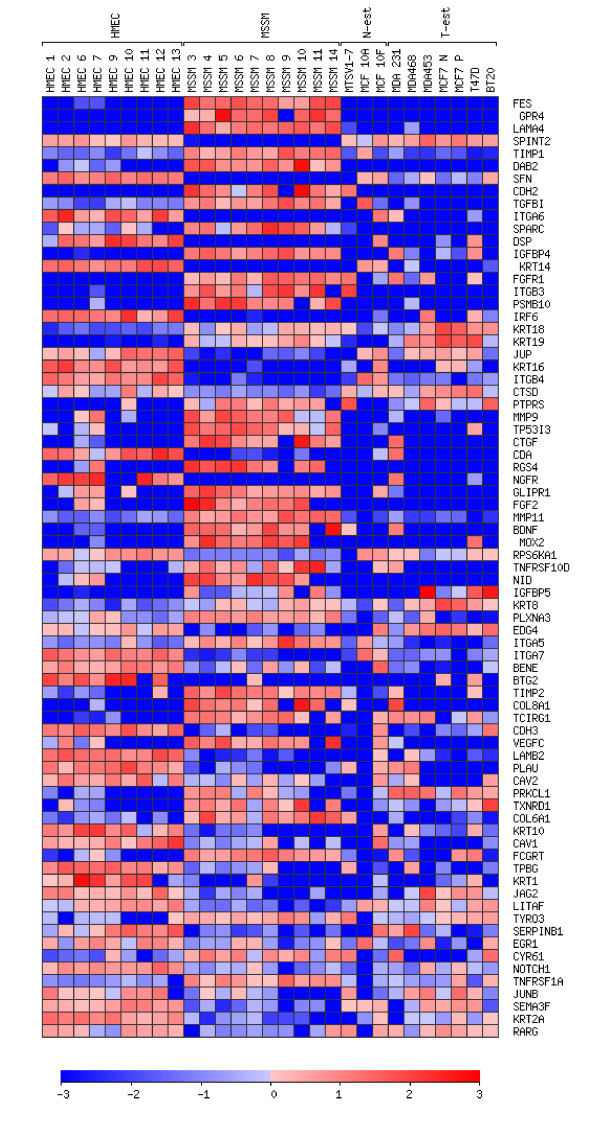
**Relative expression of all the 29 cell linestested by Atlas arrays**. Output of the Pomelo tool showing the log_2 _of the relative expression of the selected genes in each cell line to the average expression of all the cell lines. The 75 genes shown here were considered differentially expressed by SAM and by AtlasImage, with at least a 2-fold change for both programs, when we compared the HMEC and MSSM cell classes.

Of these 123 from Atlas and 101 from SAM, 75 genes were differentially expressed with at least a 2-fold change according to SAM and AtlasImage, a minimum difference in intensity of 540 units and a p-value < 0.01 (Excel). These two programs use different algorithms to calculate the ratio (AtlasImage) and fold (SAM) values, so the outputs are not exactly the same. Fig. 3 shows the relative expressions of these 75 genes in all the cell lines analyzed by the arrays using the software Pomelo Tool (FDR and p-values for each gene in Additional file 7).

Some genes showed a broad range of expression values among cell lines belonging to the same group: one or two individual cell lines over-expressed the gene (>5 fold the average of the cell class), while others in the same class gave no signal or were barely above background. Since we were looking for genes that could enable us to differentiate among types of cell lines, and hence be useful as markers for each class, we inspected the values of these genes manually. The aim was to exclude from the 75 genes in Fig 3 any that gave extreme values in 2 or more cell lines within a class; "extreme values" were those that lay in the range of the other class, or biased the average of the class in question to generate the required 2-fold change. Twenty seven up-regulated and 21 down-regulated genes remained, in addition to the cytokeratins, which are not included in Tables 3 and 4 but shown in Fig 2. Their distribution according to GO (Gene Ontology) terms by FatiGO at level 3 of biological processes is shown. These changes in gene expression stress the importance in the malignant process of both a diminished capacity for cell-cell adhesion and the remodelling of the extracellular matrix (ECM). For example: genes involved in adhesion and downstream signalling (int α4/α6, P-cadherin (CDH3), γ catenin (JUP)) or inhibitors of remodelling (SPINT-2) were down-regulated, and ECM remodelling enzymes (MMP11, TIMP2 and the cascade TIMP1-SPARC-TGFBI) were up-regulated, as shown in Tables 3 and 4 (SVG file and p-values of GOMiner in Additional files 3 and 8 respectively).

### Comparison of immortal and non-immortal breast cancer cells

Fifty genes showed significant >2-fold (according to SAM software) and >2.5-fold (according to Atlas) differences between the MSSMs and the established breast cancer cell lines. Twenty-five were up-regulated and 25 down-regulated in the established breast cancer cell lines compared to the finite life-span cultures. Most of the differentially expressed genes can be classified under the GO terms "metabolism" and "cell communication". In particular: most of the down-regulated genes seem to be related to remodelling of the extracellular matrix, cell adhesion and receptor-linked signal transduction, while the up-regulated genes are related to general signal transduction pathways and cell proliferation (Tables 5 and 6).

### Real-time PCR

To validate the differences seen in the arrays, some genes were tested by real time PCR. We selected several genes of which the expression was significantly increased or decreased, according to SAM and Atlas, for both the MSSM/HMEC and T-est/MSSM comparisons: 14-3-3σ (also called SFN), SPINT2, FES, SPARC, BIGH3 (or TGFBI), TIMP1, TIMP2, MMP11 and DAB2. Also, we analyzed some genes that were deemed significant for only one of the comparisons: NOTCH1, PLAU, CDA and SERPINB2 (or PAI2), and a few genes that were non-significant but somehow related to some of the aforementioned genes: tPA, PAI1, uPAR, DCK.

**Figure 4:**
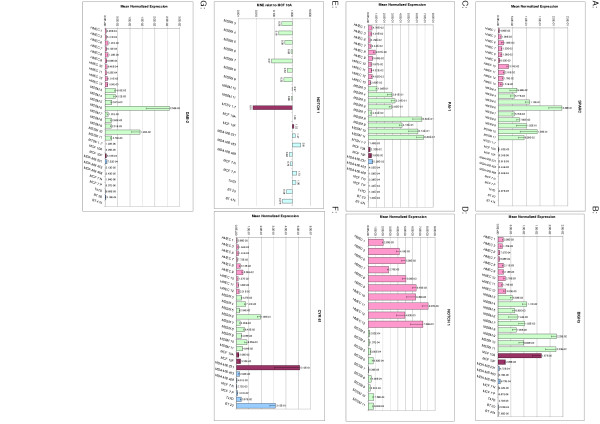
**Relative expression of some genes validated byQ-PCR**. a) Mean normalized expression (MNE) of SPARC. b) MNE of BIGH3 = TGBFI. c) MNE of PAI-1. d) MNE of NOTCH1 in HMECs and MSSMs cells. e) Relative expression, of MSSMs and established cell lines, toMCF10A. Note the difference between the two MCF7s. f) MNE of CYR61. g) MNE of DAB-2. For the complete list of validated genes, see Table S1.

It has been reported that correlation between Q-PCR and array data is highly variable [11]. It depends, in part, on the sensitivity of arrays in detecting genes with low expression levels or saturation due to very high ones. The genes we have tested showed correlations that ranged from 0.563 to 0.959. For the comparison between the normal (HMEC) and the tumour (MSSM) finite life-span cultures, the Q-PCR results for the manually curated genes (as explained above) supported the findings of the arrays in 17/17 (100%) of cases. In the T-est/MSSM evaluation, agreement between the two techniques was found in 15/17 (88.2%) of the cases (Fig. 4 and data available upon request).

One of the genes excluded from the HMEC/MSSM comparison was CYR61, which was tested by Q-PCR. Even though the overall up-regulation value for this gene was consistent (SAM: 3.53, Atlas: 3.48, Q-PCR: 3.29), its expression levels crossed over to the values of the other cell class in 4/17 cell lines, thus invalidating CYR61 as a reliable marker by itself.

## Discussion

Established breast cancer cell lines have been widely used to study signal transduction pathways, test new pharmaceuticals and determine expression profiles that might predict the metastatic capabilities of primary tumours. In many cases MCF10A has been chosen as the "normal" control, even though this cell line has been reported to possess markers for both myoepithelial and luminal phenotypes [12].

One of the controversies about the use of HMECs as controls is the probable myoepithelial origin of these cells [13] based on expression of KT14 and CD104 (ITGB4) [14,15]. The expression levels of these genes in the MCF10 cells were equivalent to those in the HMECs. Furthermore, the prediction of PAM, based on the 58 genes that discriminate between the different classes of cells used in this study, was that MCF10A was similar to HMECs. Hence, MCF10A cells would have the same limitation as controls as the HMECs. Nevertheless, HMEC cells have the advantage of not being immortalized and pooling them combines the genetic backgrounds of a large cohort.

Both classes of non-immortalized cell lines, HMEC and MSSM, are more homogeneous than the established ones (N-est and T-est), as seen in Table 2 and Additional file 1. This is probably due to the cumulative effects of the mutations accrued individually by the established cell lines during successive passages and immortalization.

As seen in Fig 3, the expression of some genes in a particular class is clearly different from the others, e.g. up-regulation of FES, MMP11, DAB2 and down-regulation of SPINT2, SFN, JUP for the MSSM cells lines. Others are more distinctive of a "state", e.g. distribution of cytokeratins and down-regulation of certain integrins (ITGA6/ITGB4, ITGA7) in all the tumour-derived cell lines. Therefore, the expression levels of these genes can be seen as specific attributes of certain classes. Their combined expression defines a profile that can be used to construct a model similar to those built by SVM and PAM for predicting the classification of an unknown cell line accurately, as shown in fig 1B.

We tested only two sub-lines of MCF-7, well-known to be highly variable [3-5], and a single sub-line of each of the other established cell lines. We recognize that other sub-lines might be different.

When the MSSMs are compared with the HMECs, several genes display differential expression in a mode consistent with previous publications, where they have been shown to be significant for malignant progression or metastasis: down-regulation of the tumour suppressor and inhibitor of mitotic phase entry 14-3-3σ (SFN) [16,17], the serine protease inhibitor SPINT2 [18] and JUP (γ-catenin) [19]; and up-regulation of FES [20] and SPARC [21]. In addition, there is the "cadherin switching" (CDH3 = P-cadherin to CDH2 = N-cadherin) and its relationship to FGFR1 and MMP9 [22,23].

SPARC expression was detected in 17/17 human breast tumour biopsies and to a lesser extent in some established cell lines [21]. It has also been associated with malignant progression and invasive potential in breast cancer [24,25], and together with MMP11 in colorectal [26] and oesophageal cancer [27]. Its over-expression increases motility and invasion [28] and induces growth inhibition [29] in established breast cancer cell lines. In addition, it has been shown to induce expression of BIGH3 and PAI-1[30]. MSSM cells showed up-regulation of SPARC, BIGH3, PAI1 and MMP11, while the T-est cells showed down-regulation of SPARC, BIGH3 and PAI1 (MDA-MB231 is an exception for the latter gene) (Fig 4A-B-C).

We also found genes of which the behaviour did not fully agree with previous descriptions, i.e. NOTCH1, CYR61 and DAB2. The many and varied functions of Notch signalling, achieved through activation or down-regulation, have been recently reviewed [31]. In the MSSM samples, NOTCH1 and its ligand JAG2 are down-regulated. In this case, this pathway is more likely to function as a tumour suppressor than an oncoprotein. This conclusion would be less clear if we had only compared established cell lines (mainly MCF10A with both MCF7s) (Fig 4D–E). CYR61 is a pro-angiogenic, secreted protein encoded by a growth factor-inducible immediate-early gene. It is over-expressed in some invasive established breast cancer cell lines and in 30–36% of primary tumours [32,33]. In this study we found only three cell lines with a truly high over-expression (MSSM6, MDA-MB231 and BT20) and five with a moderate over-expression compared to the mean expression in HMECs (between 2 and 4 fold). The same conclusion would have applied if we had considered MCF10A as a control (Fig 4F). DAB2 is considered a tumour suppressor since its expression is down-regulated in ovarian carcinomas and in some established breast cancer cell lines [34], and up-regulated during megakaryocyte differentiation [35]. Its continued expression in tumour cells led to growth inhibition or cell death [36] unless the cells were in contact with some type of basement membrane [37]. MSSM cells showed up-regulation of this gene (Fig 4G), perhaps because they grew as an attached cell line or because of their finite life-span phenotype *in vitro*.

Epithelial-mesenchymal transition (EMT) is considered a mechanism for carcinoma progression and metastasis, and the expression of vimentin (VIM) is its main marker. This view has now been extended to include whole pathways and a more complex relationship with the microenvironment of the cell [38,39]. Among the other genes regulated during EMT [40], the MSSM cells showed up-regulation of COL6A1, SPARC, CDH2 and DAB2 and down-regulation of JUP and BTG2 (Tables 3 and 4).

## Conclusion

Using arrays, we have studied 10 finite lifespan breast cancer cell lines freshly isolated from metastatic pleural or peritoneal fluids, 9 finite lifespan normal breast cell lines, 7 established breast cancer cell lines, and 3 established normal breast cell lines. We tested 1176 genes considered to be related to cancer. Within each cell class there was significant homogeneity of gene expression. Two clusters of genes distinguished the MSSMs from the HMECs. These 48 genes, which were differentially expressed by at least 2 fold, concerned cell-cell interactions and remodelling of the extracellular matrix. Fifty genes that were differentially expressed at least 2 fold between MSSMs and established breast cancer cell lines are generally considered to be involved in cell communication and metabolism. Established breast cancer cell lines have been used to model biochemical and pharmacological responses in human breast cancer; the differences from freshly isolated breast cancer lines imply they are not wholly satisfactory models.

## Abbreviations

ER = oestrogen receptor, FCS = foetal calf serum, FDR = false discovery rate, KT = keratin, MGB = mammoglobin, SVM = support vector machines, VIM = vimentin.

## Competing interests

The author(s) declare that they have no competing interests.

## Authors' contributions

MFC designed and performed all the experimental work and drafted the paper. JFH provided fundamental advice on the clinical aspects of the project and participated in the final writing of the paper. BGTP has directed the entire project and participated in the final writing of the paper.

## Pre-publication history

The pre-publication history for this paper can be accessed here:



## Supplementary Material

Additional File 1 Fig S1.doc: Pearson's correlation coefficient between cell line expression profiles Click here for file

Additional File 2 Fig S2.doc: Additional Plots and list of significant genes generated by PAM. Click here for file

Additional File 3 Fig S3: SVG (Adobe scalable vector graphics). Go Miner DAG (directed acyclical graph) view of the changed genes within the GO hierarchy. Click here for file

Additional File 4 Table S1:.doc Primer sequences and PCR-product details. Click here for file

Additional File 5 Table S2:.doc: Support Vector Machines: Validations and predictions. Click here for file

Additional File 6 Table S3:.doc: SAM output. Click here for file

Additional File 7 Table S3:.doc: False discovery rates and p-values of the Pomelo Tool applied to the comparison HMEC-MSSM. Click here for file

Additional File 8 Table S5:.xls: p-values of Go Miner. Click here for file

## Figures and Tables

**Table 1:** Cell origin and RNA expression of some frequently used markers

**cell line**	**origin ^(a)^**	**KT14**	**KT18**	**Vim**	**ER**	**ERBB2**	**ERBB3**	**MGB1**	**MGB2**
HMEC #1	mammary epithelial	+	+	++	+/-	+	+	+/-	+
HMEC #2	mammary epithelial	+	+	+	ND	ND	ND	ND	ND
HMEC #3	mammary epithelial	ND	ND	ND	-	+	+	-	+
HMEC #6	mammary epithelial	+	+	++	-	+	+	-	+
HMEC #7	mammary epithelial	+	+	++	+/-	+	+	-	+
HMEC #8	mammary epithelial	ND	ND	ND	+/-	+	+	+	+
HMEC #9	mammary epithelial	+	+	++	+	+	+	+	+
HMEC #10	mammary epithelial	+	+	++	+/-	+	+	+	+
HMEC #11	mammary epithelial	+	+	++	+	+	+	-	+
HMEC #12	mammary epithelial	+	+	+	+	+	+	-	+
HMEC #13	mammary epithelial	+	+	+	-	+	+	-	+
MSSM 3	ascites	-	+	++	+	+	+/-	-	-
MSSM 4	pleural effusion	-	+	++	-	+	-	-	+
MSSM 5	pleural effusion	-	+	++	+	+	+/-	-	+
MSSM 6	pleural effusion	-	+	++	+/-	+	+	-	+
MSSM 7	pleural effusion	+	+	++	-	+	-	+	-
MSSM 8	pleural effusion	-	+	++	+/-	+	+	+	+
MSSM 9	ascites	-	+	++	+/-	+	-	+	+
MSSM 10	pleural effusion	-	+	++	-	+	+/-	+	+
MSSM 11	pleural effusion	-	+	++	+/-	+	+	+	+
MSSM 14	ascites	-	+	++	+/-	+	-	+	+
MTSV1-7	from milk	-	+	++	-	+	+/-	-	-
MCF 10A	mammary gland, adherent	+	+	++	+/-	+	+	+	+
MCF 10F	mammary gland, floating	+	+	+/-	+	+	+	-	+
MDA-MB 231	pleural effusion	-	+	++	-	+	+	-	+
MDA-MB 453	effusion	-	+	+	-	+ ^(c)^	+	+	+
MDA-MB 468	pleural effusion	+	+	+	-	+	+	+	-
MCF 7 N	pleural effusion	-	+	+/-	+	+	+	-	+
MCF 7 P	pleural effusion	-	+	+	+	+	+	+	+
T47D	pleural effusion	-	+	-	+	+	+	+	+
BT20	prim tumor	+	+	+/-	+ ^(b)^	+	+	+	+
BT474	invasive ductal carc	ND	ND	ND	-	+	ND	+	+

Determination of the presence of mRNA for the KT and VIM markers shows the results of the array experiment. Determination for the receptors was done in triplicate by RT-PCR. +/-: 1/3 show the specific product of the PCR, -: none of the three, +: all of them, ND: not done.

(a) HMECs and established cell lines according to the providers (Clonetics, ATCC).

(b) It is known that BT20 transcribes an mRNA for this gene with a deletion in the fifth exon and hence the cell does not express the protein (ATCC).

(c) MDA-MB 453 showed a 27.2 fold up-regulation when compared to the HMECs (29.3 with MCF 10A), consistent with its amplified ERBB2 gene.

**Table 2:** Correlation between cell lines.

		median	range
**Intragroup**	HMECs	0.91	0.83–0.96
	MSSMs	0.94	0.81–0.97
	Normal-est	0.82	0.80–0.88
	Tumour-est	0.85	0.78–0.95
			
**Intergroup**	HMECs vs MSSMs	0.74	0.61–0.82
	HMECs vs Normal-est	0.85	0.69–0.93
	HMECs vs Tumour-est	0.77	0.67–0.85
	MSSMs vs Normal-est	0.77	0.68–0.87
	MSSMs vs Tumour-est	0.75	0.64–0.84
	Normal-est vs Tumour-est	0.82	0.77–0.88

Significance of HMECs or MSSMs to HMECs vs MSSMs by Wilcoxon-Mann-Whitney test two-sided, p value = p < 0.0001

**Table 3:** Gene expression of MSSM cells in relation to HMEC cells Up regulated Genes in MSSM cells

	**SAM**		**t-test**	**Atlas**			
**Name**	**Score(d)**	**Fold**	**p value**	**Ratio**	**Diff**	**Protein/gene**	**Gene ontology**

IGFBP4	9.7872	57.60	2.15E-07	47.46	5296	insulin-like growth factor binding protein 4	cell communication cellular physiological process metabolism morphogenesis regulation of cellular process
FES	8.9172	37.10	1.13E-10	31.76	1292	C-fes proto-oncogene	cell communication cellular physiological process metabolism
LAMA4	7.8262	159.92	2.95E-12	131.73	3399	laminin alpha 4 subunit	cell communication cellular physiological process regulation of cellular process
FCGRT	7.4658	6.22	1.43E-03	5.18	3425	IgG receptor FC large subunit P51	response to stimulus
FGFR1	7.3244	1925.86	7.01E-12	Up	2368	N-sam; fibroblast growth factor receptor1	cell communication metabolism morphogenesis
MMP11	6.7459	4.79	4.30E-08	4.67	29606	matrix metalloproteinase 11; stromelysin 3	metabolism morphogenesis
TIMP1	6.3100	4.22	3.66E-07	4.15	30855	metalloproteinase inhibitor 1	cellular physiological process metabolism regulation of cellular process
TIMP2	6.3100	4.22	6.14E-04	5.64	1713	tissue inhibitor of metalloproteinases 2	cellular physiological process
PTPRS	6.2185	11.57	5.03E-05	12.56	705	protein tyrosine phosphatase sigma	cell communication metabolism
TGFBI	6.0130	3.99	1.71E-06	4.05	6759	BIGH3	cell communication cellular physiological process regulation of cellular process response to stimulus
ITGB3	5.5711	71.88	6.47E-09	57.56	1527	integrin beta 3; CD61 antigen	cell communication
TCIRG1	5.3497	15.08	9.83E-05	14.66	1257	specific 116-kDa vacuolar proton pump	cellular physiological process regulation of cellular process response to stimulus
TNFRSF1A	5.3392	2.97	2.29E-06	2.90	1875	tumor necrosis factor receptor 1	cell communication cellular physiological process regulation of cellular process
MOX2	5.1746	42.04	3.61E-05	39.85	777	OX2 membrane glycoprotein	Gen with GO but NOT at level 3 biological process
TP53I3	4.9520	6.53	8.84E-05	5.73	1225	PIG3	Gen with GO but NOT at level 3 biological process
BDNF	4.8902	24.15	7.48E-05	21.05	762	brain-derived neurotrophic factor	morphogenesis
TYRO3	4.3661	3.23	9.88E-03	2.93	1425	tyrosine-protein kinase receptor tyro3 precursor; rse; sky; dtk	cell communication metabolism
CTRL	4.2166	25.91	4.47E-05	19.65	1063	Chymotrypsin-like protease CTRL-1	metabolism
SPARC	4.1228	4.21	5.59E-04	4.30	2840	secreted protein acidic and rich in cysteine; osteonectin	morphogenesis
GPR4	3.8917	604.85	1.01E-05	Up	727	probable G protein-coupled receptor	cell communication
DAB2	3.8635	8.91	1.48E-05	8.36	1339	mitogen-responsive phosphoprot. DOC2	cellular physiological process
COL6A1	3.7320	4.57	3.96E-04	4.44	3145	collagen 6 alpha 1 subunit	cell communication cellular physiological process
CDH2	3.7267	910.06	1.16E-05	Up	1162	N-cadherin; cadherin 2	cell communication
CTGF	3.7190	19.64	2.31E-05	17.28	1091	connective tissue growth factor	cell communication cellular physiological process metabolism morphogenesis regulation of cellular process response to stimulus
ITGA5	3.5646	3.49	2.47E-05	3.36	3815	integrin alpha 5	cell communication regulation of cellular process
IGFBP5	3.4913	27.63	2.39E-04	21.90	1045	insulin-like growth factor binding protein 5	cell communication cellular physiological process morphogenesis
TNFRSF10D	3.2716	6.15	4.92E-04	5.25	1514	decoy receptor 2	cellular physiological process

**Table 4:** Gene expression of MSSM cells in relation to HMEC cells Down regulated Genes in MSSM cells.

	**SAM**		**t-test**	**Atlas**			
**Name**	**Score(d)**	**Fold**	**p value**	**Ratio**	**Diff**	**Protein/gene**	**Gene ontology**

SFN	-18.5106	-134.41	2.71E-08	-76.89	-14875	14-3-3 protein sigma; stratifin	cell communication cellular physiological process metabolism
SPINT2	-14.6598	-37.30	4.10E-07	-39.72	-5653	bikunin; hepatocyte growth factor activator inhibitor 2	cellular physiological process
LAMB2	-11.3177	-12.53	8.94E-06	-12.09	-5510	laminin beta 2 subunit precursor	cell communication
ITGA7	-11.0881	-13.03	3.24E-05	-12.06	-12577	integrin alpha 7B precursor	cell communication cellular physiological process morphogenesis
CDH3	-8.9105	-12.11	3.62E-05	-15.23	-4283	cadherin 3; P-cadherin	cell communication response to stimulus
PLAU	-7.9173	-10.37	2.18E-05	-22.29	-2512	urokinase-type plasminogen activator	cell communication cellular physiological process metabolism response to stimulus
ITGB4	-7.9148	-22.09	2.86E-05	-20.11	-16722	integrin beta 4; CD104 antigen	cell communication
TPBG	-7.6578	-5.42	2.34E-04	-4.84	-1922	5T4 oncofetal antigen precursor	cell communication cellular physiological process
RPS6KA1	-7.5622	-3.37	5.64E-08	-3.21	-3450	ribosomal protein S6 kinase alpha1	cell communication metabolism
EDG4	-7.2225	-4.69	6.71E-04	-4.45	-1208	G protein-coupled receptor	cell communication
IRF6	-6.7854	-120.19	4.26E-11	137.08	-1905	interferon regulatory factor 6	metabolism
LITAF	-6.7240	-3.73	1.49E-03	-3.53	-2311	PIG7	cell communication metabolism regulation of cellular process
SEMA3F	-6.4547	-3.55	1.90E-04	-2.68	-1510	semaphorin	Gen with GO but NOT at level 3 biological process
NOTCH1	-6.0498	-2.95	7.96E-04	-2.86	-1883	neurogenic locus notch protein homolog 1	metabolism response to stimulus
ITGA6	-5.9332	1052.63	6.90E-07	Down	-1273	integrin alpha 6 precursor	cell communication cellular physiological process
DSP	-5.9015	1190.48	8.57E-07	Down	-1463	desmoplakin I & II	morphogenesis
CDA	-5.4364	-21.03	4.64E-07	-18.79	-3523	cytidine deaminase	metabolism
JUP	-5.3936	-6.77	9.66E-07	-6.47	-6165	junction plakoglobin; desmoplakin III	cell communication
JAG2	-5.2949	-4.22	1.20E-03	-4.30	-1841	jagged2	cell communication cellular physiological process morphogenesis regulation of cellular process response to stimulus
SERPINB1	-4.8852	-6.11	1.38E-04	-5.46	-1960	leukocyte elastase inhibitor	Gen with GO but NOT at level 3 biological process
BTG2	-4.8636	-24.34	3.84E-05	-11.74	-752	btg protein precursor; NGF- inducible anti-proliferative protein PC3	cellular physiological process metabolism regulation of cellular process response to stimulus

Genes ordered according to their SAM scores and distribution of the genes according the GO terms using FatiGo

**Table 5:** Gene expression of T-est cells in relation to MSSM cells 25 Significant Up-regulated Genes in T-est cells

	**SAM**		**Atlas**			
**Name**	**Score(d)**	**Fold**	**Ratio**	**Diff**	**Protein/gene**	**Gene ontology**

SPINT2	9.3646	51.350	65.437	7668	bikunin; hepatocyte growth factor activator inhibitor 2	cellular physiological process
PSME1	7.7824	3.034	3.630	3419	interferon-gamma IEF SSP 5111	response to stimulus
SFN	6.5917	37.394	25.868	3954	14-3-3 protein sigma; stratifin	cell communication cellular physiological process metabolism
JUP	6.2887	4.779	5.825	4405	junction plakoglobin; desmoplakin III	cell communication
TFAP2C	6.0176	89.861	108.133	1607	transcription factor erf-1; AP2 gamma transc factor	cell communication metabolism
SLC25A5	5.6914	2.028	2.512	4761	ADP/ATP carrier protein	cellular physiological process
GSTZ1	5.5683	11.704	13.413	1986	glutathione transferase zeta 1	metabolism
TBRG4	5.4262	2.362	2.859	3099	cell cycle progression 2 protein (CPR2)	Gen with GO but NOT at level 3 biological process
EDG4	5.3459	7.169	8.197	2044	G protein-coupled receptor EDG4	cell communication
RPS6KA1	5.0882	2.030	2.528	1933	ribosomal S6 kinase 1	cell communication metabolism
CTSD	4.9896	3.430	4.120	6462	cathepsin D precursor	metabolism
HDGF	4.8236	2.112	2.590	2185	hepatoma-derived growth factor	cell communication cellular physiological process
PCTK1	4.5911	2.202	2.648	2444	serine/threonine-protein kinase PCTAIRE 1	cellular physiological process metabolism
LITAF	4.4875	3.318	3.923	2166	PIG7	cell communication metabolism regulation of cellular process
PTPN6	4.4289	15.939	12.218	1391	protein-tyrosine phosphatase1C	cell communication cellular physiological process metabolism
RFC2	4.3235	2.469	2.800	2036	replication factor C 40-kDa subunit (RFC40)	cellular physiological process metabolism
RARG	4.3142	2.640	3.020	1085	retinoic acid receptor gamma 1	metabolism
ADRBK1	4.2331	3.963	4.121	1211	G protein-coupled receptor kinase 2 (GRK2)	cell communication metabolism
CKS1B	4.2166	3.370	4.301	3341	cyclin-dependent kinase regulatory subunit 1	Gen with GO but NOT at level 3 biological process
RPS21	4.1856	2.210	2.674	2531	ribosomal protein S21	metabolism
AURKB	4.1129	4.052	5.168	1980	aurora- & IPL1-like protein kinase1; ARK2	cellular physiological process metabolism
PPP2R4	3.9781	3.481	4.068	2433	phosphotyrosyl phosphatase activator	metabolism
FASN	3.6731	2.953	3.615	4590	fatty acid synthase	metabolism
GDF15	3.6008	5.032	5.878	10268	macrophage inhibitory cytokine 1 (MIC1)	Gen with GO but NOT at level 3 biological process
MAPK13	3.5903	5.374	6.228	1652	stress-activated protein kinase 4 (SAPK4)	cell communication cellular physiological process metabolism response to stimulus

Genes ordered according to their SAM scores and distribution of the genes according the GO terms using FatiGo

**Table 6:** Gene expression of T-est cells in relation to MSSM cells 25 Significant Down-regulated Genes in T-est cells

	**SAM**		**Atlas**			
**Name**	**Score(d)**	**Fold**	**Ratio**	**Diff.**	**Protein/gene**	**Gene ontology**

FES	-7.9514	-1063.830	Down	-1081	C-fes proto-oncogene	cell communication cellular physiological process metabolism
VIM	-7.0022	-5.070	-3.698	-9614	vimentin	Gen with GO but NOT at level 3 biological process
TGFBI	-6.6491	-22.619	-17.180	-6844	BIGH3	cell communication cellular physiological process regulation of cellular process response to stimulus
BMP1	-6.5296	-12.975	-9.085	-1609	bone morphogenetic prot. 1+ procollagen C-proteinase	metabolism
MLH1	-6.4271	-9.043	-6.664	-1552	mutL protein homolog; DNA mismatch repair protein	cellular physiological process metabolism response to stimulus
LAMA4	-6.3501	-24.913	-19.264	-2630	laminin alpha 4 subunit precursor	cell communication
MMP11	-6.2022	-6.609	-5.019	-24432	matrix metalloproteinase 11; stromelysin 3	metabolism
TIMP1	-6.1061	-6.355	-4.793	-26048	metalloproteinase inhibitor 1	cellular physiological process metabolism regulation of cellular process
PLXNA3	-5.9530	-3.489	-2.845	-2334	transmembrane protein sex precursor	Gen with GO but NOT at level 3 biological process
TIMP2	-5.1069	-6.041	-4.092	-1274	tissue inhibitor of metalloproteinases 2	cellular physiological process
ITGB8	-5.0533	-12.188	-8.712	-2414	integrin beta 8	cell communication
ITGB3	-4.8610	-1282.051	Down	-1259	integrin beta 3	cell communication
SPARC	-4.7998	-2857.143	Down	-2997	secreted protein acidic and rich in cysteine precursor; osteonectin	physiological process
FN1	-4.5334	-50.633	-34.784	-10608	fibronectin precursor	cell communication cellular physiological process metabolism response to stimulus
MMP9	-4.5288	-12.126	-7.859	-1365	matrix metalloproteinase 9	metabolism
HSPG2	-4.3027	-1149.425	Down	-1164	heparan sulfate proteoglycan	cell communication
MMP14	-4.0581	-5.566	-3.840	-2454	matrix metalloproteinase 14; MT- MMP1	metabolism
ITGA5	-3.9413	-11.151	-7.838	-3836	integrin alpha 5	cell communication
MT3	-3.9295	-7.365	-5.866	-9946	metallothionein-III	cellular physiological process metabolism regulation of cellular process response to stimulus
DAB2	-3.8058	-1190.476	Down	-1232	mitogen-responsive phosphoprotein DOC2	cellular physiological process
SERPINB2	-3.6797	-1818.182	Down	-1773	placental plasminogen activator inhibitor 2 (PAI-2); urokinase inhibitor	cellular physiological process regulation of cellular process
IGFBP6	-3.6326	-12.402	-8.758	-1668	insulin-like growth factor binding protein 6 precursor	cell communication cellular physiological process regulation of cellular process
COL6A1	-3.6304	-8.586	-6.311	-2767	collagen 6 alpha 1 subunit (COL6A1)	cell communication cellular physiological process
IL6	-3.4586	-10.628	-7.933	-2586	interleukin-6 precursor	cell communication cellular physiological process regulation of cellular process response to stimulus
LIF	-3.4166	-5.400	-3.940	-1085	leukemia inhibitory factor precursor	cell communication cellular physiological process regulation of cellular process response to stimulus

Genes ordered according to their SAM scores and distribution of the genes according the GO terms using FatiGo
