# Supplementary material for: Transcription profiles of non-immortalized breast cancer cell lines
Source: BMC Cancer. 2006 Apr 20;6:99. doi: 10.1186/1471-2407-6-99 (PMC1524972; doi:10.1186/1471-2407-6-99)
Supplement: Additional File 1 — Fig S1.doc: Pearson's correlation coefficient between cell line expression profiles [file 1471-2407-6-99-S1.doc]

Fig. S1: Pearson’s correlation coefficients

1 2 3 4 5 6 7 8 9 101112131415161718192021222324252627

1 2 3 4 5 6 7 8 9 10 11 12 13 14 15 16 17 18 19 20 21 22 23 24 25 26 27
