# Supplementary material for: Transcription profiles of non-immortalized breast cancer cell lines
Source: BMC Cancer. 2006 Apr 20;6:99. doi: 10.1186/1471-2407-6-99 (PMC1524972; doi:10.1186/1471-2407-6-99)
Supplement: Additional File 2 — Fig S2.doc: Additional Plots and list of significant genes generated by PAM. [file 1471-2407-6-99-S2.doc]

**Fig S2**: Plots and List of significant genes generated by PAM

**Training Confusion Matrix (Threshold=3.5)**

**True\Predicted HMEC MSSM T est Class Error rate**

**HMEC** 8 0 0 0

**MSSM** 0 9 0 0

**T est** 0 0 7 0

**CV Confusion Matrix (Threshold=3.58143)**

**True\Predicted HMEC MSSM T est Class Error rate**

**HMEC** 8 0 0 0

**MSSM** 0 9 0 0

**T est** 0 0 7 0

**Settings**

**Offset Quantile** 50 **Offset Value** 336.6217127

**Contrast** both **RNG Seed** 362436069

**Prior Distribution (Sample Prior)**

**Class** HMEC MSSM T est

**Prob.** 0.3333 0.375 0.291666667

| List of Significant Genes for Threshold = 3.5 | | | |
| --- | --- | --- | --- |
| **Prior Distribution (Sample Prior)** | | | |
| **Class** | HMEC | MSSM | T est |
| **Prob.** | 0.333333333 | 0.375 | 0.291666667 |
|  |  |  |  |
| name | HMEC score | **MSSM score** | **T est score** |
| [KRT14](http://genome-www4.stanford.edu/cgi-bin/SMD/source/sourceResult?choice=Gene&option=Name&criteria= KRT14) | 2.7374 | -1.1145 | -0.551 |
| [SFN](http://genome-www4.stanford.edu/cgi-bin/SMD/source/sourceResult?choice=Gene&option=Name&criteria=SFN) | 2.6042 | -1.7627 | 0 |
| [ITGA7](http://genome-www4.stanford.edu/cgi-bin/SMD/source/sourceResult?choice=Gene&option=Name&criteria=ITGA7) | 2.1964 | -0.9563 | -0.1361 |
| [LAMB2](http://genome-www4.stanford.edu/cgi-bin/SMD/source/sourceResult?choice=Gene&option=Name&criteria=LAMB2) | 1.5719 | -0.4638 | -0.0556 |
| [ITGB4](http://genome-www4.stanford.edu/cgi-bin/SMD/source/sourceResult?choice=Gene&option=Name&criteria=ITGB4) | 1.522 | -0.5852 | 0 |
| [MMP11](http://genome-www4.stanford.edu/cgi-bin/SMD/source/sourceResult?choice=Gene&option=Name&criteria=MMP11) | -0.2578 | 1.4234 | -0.4532 |
| [TIMP1](http://genome-www4.stanford.edu/cgi-bin/SMD/source/sourceResult?choice=Gene&option=Name&criteria=TIMP1) | -0.1465 | 1.3134 | -0.439 |
| [SPINT2](http://genome-www4.stanford.edu/cgi-bin/SMD/source/sourceResult?choice=Gene&option=Name&criteria=SPINT2) | 0 | -1.2859 | 0.7689 |
| [TGFBI](http://genome-www4.stanford.edu/cgi-bin/SMD/source/sourceResult?choice=Gene&option=Name&criteria=TGFBI) | 0 | 1.0451 | -0.4795 |
| [IL1B](http://genome-www4.stanford.edu/cgi-bin/SMD/source/sourceResult?choice=Gene&option=Name&criteria=IL1B) | 0.9946 | -0.0482 | 0 |
| [LAMA4](http://genome-www4.stanford.edu/cgi-bin/SMD/source/sourceResult?choice=Gene&option=Name&criteria=LAMA4) | -0.0861 | 0.8419 | 0 |
| [KRT2A](http://genome-www4.stanford.edu/cgi-bin/SMD/source/sourceResult?choice=Gene&option=Name&criteria=KRT2A) | 0.7808 | -0.2494 | 0 |
| [VIM](http://genome-www4.stanford.edu/cgi-bin/SMD/source/sourceResult?choice=Gene&option=Name&criteria=VIM) | 0 | 0.7361 | -0.7787 |
| [PSME1](http://genome-www4.stanford.edu/cgi-bin/SMD/source/sourceResult?choice=Gene&option=Name&criteria=PSME1) | 0 | 0 | 0.7271 |
| [IGFBP4](http://genome-www4.stanford.edu/cgi-bin/SMD/source/sourceResult?choice=Gene&option=Name&criteria=IGFBP4) | -0.34 | 0.6015 | 0 |
| [CDH3](http://genome-www4.stanford.edu/cgi-bin/SMD/source/sourceResult?choice=Gene&option=Name&criteria=CDH3) | 0.5714 | -0.0863 | 0 |
| [FN1](http://genome-www4.stanford.edu/cgi-bin/SMD/source/sourceResult?choice=Gene&option=Name&criteria=FN1) | 0 | 0.0876 | -0.4497 |
| [KRT10](http://genome-www4.stanford.edu/cgi-bin/SMD/source/sourceResult?choice=Gene&option=Name&criteria=KRT10) | 0.4347 | 0 | 0 |
| [RPS6KA1](http://genome-www4.stanford.edu/cgi-bin/SMD/source/sourceResult?choice=Gene&option=Name&criteria=RPS6KA1) | 0.4255 | -0.3264 | 0 |
| [TPBG](http://genome-www4.stanford.edu/cgi-bin/SMD/source/sourceResult?choice=Gene&option=Name&criteria=TPBG) | 0.3812 | 0 | 0 |
| [KRT19](http://genome-www4.stanford.edu/cgi-bin/SMD/source/sourceResult?choice=Gene&option=Name&criteria=KRT19) | -0.3457 | 0 | 0.3702 |
| [BENE](http://genome-www4.stanford.edu/cgi-bin/SMD/source/sourceResult?choice=Gene&option=Name&criteria=BENE) | 0.3278 | 0 | 0 |
| [PLXNA3](http://genome-www4.stanford.edu/cgi-bin/SMD/source/sourceResult?choice=Gene&option=Name&criteria=PLXNA3) | 0 | 0.321 | -0.0145 |
| [SPARC](http://genome-www4.stanford.edu/cgi-bin/SMD/source/sourceResult?choice=Gene&option=Name&criteria=SPARC) | 0 | 0.3191 | 0 |
| [FES](http://genome-www4.stanford.edu/cgi-bin/SMD/source/sourceResult?choice=Gene&option=Name&criteria=FES) | 0 | 0.2916 | 0 |
| [CDA](http://genome-www4.stanford.edu/cgi-bin/SMD/source/sourceResult?choice=Gene&option=Name&criteria=CDA) | 0.2863 | 0 | 0 |
| [ITGA5](http://genome-www4.stanford.edu/cgi-bin/SMD/source/sourceResult?choice=Gene&option=Name&criteria=ITGA5) | 0 | 0.2454 | 0 |
| [JUP](http://genome-www4.stanford.edu/cgi-bin/SMD/source/sourceResult?choice=Gene&option=Name&criteria=JUP) | 0.1363 | -0.2314 | 0 |
| [MMP14](http://genome-www4.stanford.edu/cgi-bin/SMD/source/sourceResult?choice=Gene&option=Name&criteria=MMP14) | 0 | 0 | -0.2271 |
| [TIMP2](http://genome-www4.stanford.edu/cgi-bin/SMD/source/sourceResult?choice=Gene&option=Name&criteria=TIMP2) | 0 | 0.2258 | 0 |
| [TBRG4](http://genome-www4.stanford.edu/cgi-bin/SMD/source/sourceResult?choice=Gene&option=Name&criteria=TBRG4) | 0 | 0 | 0.2099 |
| [PLAU](http://genome-www4.stanford.edu/cgi-bin/SMD/source/sourceResult?choice=Gene&option=Name&criteria=PLAU) | 0.2048 | 0 | 0 |
| [LITAF](http://genome-www4.stanford.edu/cgi-bin/SMD/source/sourceResult?choice=Gene&option=Name&criteria=LITAF) | 0 | -0.1955 | 0 |
| [CKS1B](http://genome-www4.stanford.edu/cgi-bin/SMD/source/sourceResult?choice=Gene&option=Name&criteria=CKS1B) | 0 | 0 | 0.1793 |
| [COL6A1](http://genome-www4.stanford.edu/cgi-bin/SMD/source/sourceResult?choice=Gene&option=Name&criteria=COL6A1) | 0 | 0.1735 | 0 |
| [ITGB3](http://genome-www4.stanford.edu/cgi-bin/SMD/source/sourceResult?choice=Gene&option=Name&criteria=ITGB3) | 0 | 0.1656 | 0 |
| [ITGA3](http://genome-www4.stanford.edu/cgi-bin/SMD/source/sourceResult?choice=Gene&option=Name&criteria=ITGA3) | 0.1646 | 0 | 0 |
| [GSTO1](http://genome-www4.stanford.edu/cgi-bin/SMD/source/sourceResult?choice=Gene&option=Name&criteria=GSTO1) | 0 | 0.1554 | 0 |
| [MLH1](http://genome-www4.stanford.edu/cgi-bin/SMD/source/sourceResult?choice=Gene&option=Name&criteria=MLH1) | 0 | 0 | -0.1495 |
| [AURKB](http://genome-www4.stanford.edu/cgi-bin/SMD/source/sourceResult?choice=Gene&option=Name&criteria=AURKB) | 0 | 0 | 0.1381 |
| [FCGRT](http://genome-www4.stanford.edu/cgi-bin/SMD/source/sourceResult?choice=Gene&option=Name&criteria=FCGRT) | 0 | 0.1323 | 0 |
| [ITGB8](http://genome-www4.stanford.edu/cgi-bin/SMD/source/sourceResult?choice=Gene&option=Name&criteria=ITGB8) | 0 | 0.1283 | -0.0786 |
| [NOTCH1](http://genome-www4.stanford.edu/cgi-bin/SMD/source/sourceResult?choice=Gene&option=Name&criteria=NOTCH1) | 0.1087 | 0 | 0 |
| [IRF6](http://genome-www4.stanford.edu/cgi-bin/SMD/source/sourceResult?choice=Gene&option=Name&criteria=IRF6) | 0.1026 | 0 | 0 |
| [FGFR1](http://genome-www4.stanford.edu/cgi-bin/SMD/source/sourceResult?choice=Gene&option=Name&criteria=FGFR1) | 0 | 0.0886 | 0 |
| [KRT18](http://genome-www4.stanford.edu/cgi-bin/SMD/source/sourceResult?choice=Gene&option=Name&criteria=KRT18) | -0.0239 | 0 | 0.079 |
| [TFAP2C](http://genome-www4.stanford.edu/cgi-bin/SMD/source/sourceResult?choice=Gene&option=Name&criteria=TFAP2C) | 0 | 0 | 0.0709 |
| [EDG4](http://genome-www4.stanford.edu/cgi-bin/SMD/source/sourceResult?choice=Gene&option=Name&criteria=EDG4) | 0 | -0.0696 | 0 |
| [ERCC1](http://genome-www4.stanford.edu/cgi-bin/SMD/source/sourceResult?choice=Gene&option=Name&criteria=ERCC1) | 0.0651 | 0 | 0 |
| [PPP2R4](http://genome-www4.stanford.edu/cgi-bin/SMD/source/sourceResult?choice=Gene&option=Name&criteria=PPP2R4) | 0 | 0 | 0.0444 |
| [TNFRSF1A](http://genome-www4.stanford.edu/cgi-bin/SMD/source/sourceResult?choice=Gene&option=Name&criteria=TNFRSF1A) | 0 | 0.0331 | 0 |
| [DSP](http://genome-www4.stanford.edu/cgi-bin/SMD/source/sourceResult?choice=Gene&option=Name&criteria=DSP) | 0.0261 | 0 | 0 |
| [MMP9](http://genome-www4.stanford.edu/cgi-bin/SMD/source/sourceResult?choice=Gene&option=Name&criteria=MMP9) | 0 | 0.0243 | 0 |
| [ITGA6](http://genome-www4.stanford.edu/cgi-bin/SMD/source/sourceResult?choice=Gene&option=Name&criteria=ITGA6) | 0.0158 | 0 | 0 |
| [SERPINB2](http://genome-www4.stanford.edu/cgi-bin/SMD/source/sourceResult?choice=Gene&option=Name&criteria=SERPINB2) | 0 | 0.0145 | 0 |
| [CTSD](http://genome-www4.stanford.edu/cgi-bin/SMD/source/sourceResult?choice=Gene&option=Name&criteria=CTSD) | 0 | -0.0066 | 0 |
| [CDKN1A](http://genome-www4.stanford.edu/cgi-bin/SMD/source/sourceResult?choice=Gene&option=Name&criteria=CDKN1A) | 0.0054 | 0 | 0 |
| [IL6](http://genome-www4.stanford.edu/cgi-bin/SMD/source/sourceResult?choice=Gene&option=Name&criteria=IL6) | 0 | 0.0053 | 0 |
