# Supplementary material for: Transcription profiles of non-immortalized breast cancer cell lines
Source: BMC Cancer. 2006 Apr 20;6:99. doi: 10.1186/1471-2407-6-99 (PMC1524972; doi:10.1186/1471-2407-6-99)
Supplement: Additional File 4 — Table S1:.doc Primer sequences and PCR-product details. [file 1471-2407-6-99-S4.doc]

# Table S1: list of primers

# Gene Acc # primers (5’-3’) frag.(bp) Tm frag. chrom

G3PDH X01677 G3PDH-F GTGAAGGTCGGAGTCAACGGA 300 83 12

G3PDH-3 ggtgaagacgccagtggactc (a) 2154bp

β-ACTIN X00351 actin-5 CCACTGGCATCGTGATGGAC 428 87 7

actin-3 GCGGATGTCCACGTCACACT 523 bp

14-3-3 σ= SFN XM059066 Stra F TAGGCGCTGTTCTTGCTCCAAA 349 87 1

Stra R TTCCCTCAATCTCGGTCTTGCAC 349bp

Cyr61 AF031385 Cyr F TGAGTGCCGCCTTGTGAAAGAA 316 85 1

Cyr R ATGCGGGCAGTTGTAGTTGCATT 431bp

DAB-2 NM001343 DAB-2-F CTCACCACATGACTCCATAGCC 309 85 5

DAB-2-R GGGGACTGATTGAAGACCAAAG 5481bp

TIMP1 X03124 TIMP-1-F TGGACTCTTGCACATCACTACCTG 349 87 X

TIMP-1-R GGAAGAAAGATGGGAGTGGGAACA 1202bp

TIMP2 J05593 TIMP-2-F AGGAAGTGGACTCTGGAAACGACA 310 85 17

TIMP-2-R ATCTTGCACTCGCAGCCCATCT 16370bp

MMP11 X57766 MMP-11-F CTAAAGGTATGGAGCGATGTGAC 327 87 22

=stromelysin3 MMP-11-R TGGGTAGCGAAAGGTGTAGAAG (b) 541bp

TGFBI M77349 BIGH-3-F TGTGTCCACAGCCATTGACCTT 384 85 5

= BIGH3 BIGH-3-R AAGCGATTGTCTCCCTTCAGGA 1842

SPARC J03040 SPARC-F ggatgaggacaacaaccttctg 396 86.5 5

SPARC-R aatccggtactgtggaaggagt 3966bp

FES X52192 FES-F ttcctttgctcatcgaccacct 352 86.5 15

FES-R tgcacaagctccatgacgatgt 1607bp

NOTCH1 M73980 NOT-1-F AGATGCCAACATCCAGGACAAC 309 89 9

NOT-1-R TCTTTGTTAGCCCCGTTCTTCA 1716

SPINT2= HAI-2/ U78095 HAI-2 232 TACCTGACCAAGGAGGAGTGC 408 86 19

NM021102 HAI-2 616 AGGAAGAGGATCAACACCATCAC (a) 8135bp

SERPINB2 M18082 PAI-2-F AACAAGGGACGGGCCAATTTCT 305 83.5 18

= PAI-2 PAI-2-R TTCTGAGGCACACAGCTCATCT 305bp

PAI1 X04429 PAI1-F GAAACCCAGCAGCAGATTCAAGCA 361 85.5 7

SERPINE1 PAI1-R CGTTGAAGTAGAGGGCATTCACCA 3337bp

PLAU M15476 PLAU-F ATTGCCTTGCTGAAGATCCGTTCC 352 84.5 10

PLAU-R TTCCAGTCAAAGTCATGCGGCCTT 1684bp

tPA M15518 tPA-F AACGATGGCCGCATGACTTTGGT 379 84.5 8

tPA-R CTGGAGAGGCTAGTGTGCATTCAT 379bp

uPAR NM002659 uPAR-F TGTAAGACCAACGGGGATTG 405 86 19

uPAR-R ATCCTTTGGACGCCCTTCTT 12120

CDA L27943 CDA-F AGAAGGGTACAAGGATTTCAGGGCA 394 85.5 1

CDA-R TCTAAGTCCCAAGGCAGGTTGCTA 13727

DCK- M60527 DCK-F TTTGTCTTCCTCAGCAGGTTGGC 366 78 4

DCK-R GCAGAACCCTACAAAGCAAAGCA 366bp

ERBB2 M11730 HER-2-F TTTGGCACAGTCTACAAGGGCA 380 86.5 17

HER2/neu HER-2-R TTGGGACTCTTGACCAGCACGTT 1494bp

ERBB3 M29366 ERB-3-F TTGGCTCGGGTGTCTTTGGAACT 373 84.5 12

ERB-3-R TTTCGGGCAGCCAGGTTTCTAT 1250bp

ER X03635 ER-F GCCATTGCCTAGCTTGCCGTAAT 388 79.5 6

ER-R TCCCAGTACCCACAGTCCATCTCA 388bp

MGB1 U33147 MGB1F TGAACACCGACAGCAGCAG 367 60 11

mgb1r TCCGTAGTTGGTTTCTCACC (c) 60 2857

MGB2 AF071219 MGB2F ACTCCTGGAGGACATGGTTGA 245 64 11

MGB2R TCTGAGCCAAACGCCTTGGGT (d) 66 3370

(a): [Hamasuna R, Kataoka H, Meng JY, Itoh H, Moriyama T, Wakisaka S, Koono M.](http://www.ncbi.nlm.nih.gov:80/entrez/query.fcgi?cmd=Retrieve&db=PubMed&list_uids=11433397&dopt=Abstract) . Reduced expression of hepatocyte growth factor activator inhibitor type-2/placental bikunin (HAI-2/PB) in human glioblastomas: implication for anti-invasive role of HAI-2/PB in glioblastoma cells. Int J Cancer. 2001 Aug 1;93(3):339-45.

(b): [Giambernardi TA, Grant GM, Taylor GP, Hay RJ, Maher VM, McCormick JJ, Klebe RJ.](http://www.ncbi.nlm.nih.gov:80/entrez/query.fcgi?cmd=Retrieve&db=PubMed&list_uids=9550265&dopt=Abstract) Overview of matrix metalloproteinase expression

in cultured human cells. Matrix Biol. 1998 Mar;16(8):483-96.

(c): [Fiegl M, Haun M, Massoner A, Krugmann J, Muller-Holzner E, Hack R, Hilbe W, Marth C, Duba HC, Gastl G, Grunewald K.](http://www.ncbi.nlm.nih.gov/entrez/query.fcgi?cmd=Retrieve&db=pubmed&dopt=Abstract&list_uids=14752070) Combination of cytology, fluorescence in situ hybridization for aneuploidy, and reverse-transcriptase polymerase chain reaction for human mammaglobin/mammaglobin B expression improves diagnosis of malignant effusions. J Clin Oncol. 2004 Feb 1;22(3):474-83.

(d): [Ouellette RJ, Richard D, Maicas E.](http://www.ncbi.nlm.nih.gov/entrez/query.fcgi?cmd=Retrieve&db=pubmed&dopt=Abstract&list_uids=15151203) RT-PCR for mammaglobin genes, MGB1 and MGB2, identifies breast cancer micrometastases in sentinel lymph nodes. Am J Clin Pathol. 2004 May;121(5):637-43.
