# Supplementary material for: Transcription profiles of non-immortalized breast cancer cell lines
Source: BMC Cancer. 2006 Apr 20;6:99. doi: 10.1186/1471-2407-6-99 (PMC1524972; doi:10.1186/1471-2407-6-99)
Supplement: Additional File 5 — Table S2:.doc: Support Vector Machines: Validations and predictions. [file 1471-2407-6-99-S5.doc]

Table S2 : Support Vector Machines at the Bioinformatics Unit, CNIO

Results generated by 24 - fold Cross Validation:

| **Sample:** | **Real class:** | **Prediction:** | |
| --- | --- | --- | --- |
| MSSM_5 | MSSM | MSSM | 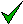 |
| HMEC#11 | HMEC | HMEC | 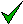 |
| HMEC#2 | HMEC | HMEC | 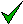 |
| MSSM_7 | MSSM | MSSM | 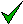 |
| T47D | T est | T est | 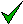 |
| MSSM_10 | MSSM | MSSM | 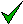 |
| MSSM_8 | MSSM | MSSM | 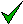 |
| HMEC#7 | HMEC | HMEC | 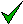 |
| BT20 | T est | T est | 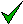 |
| MSSM_9 | MSSM | MSSM | 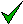 |
| HMEC#9 | HMEC | HMEC | 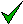 |
| HMEC#6 | HMEC | HMEC | 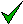 |
| MSSM_4 | MSSM | MSSM | 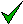 |
| HMEC#10 | HMEC | HMEC | 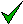 |
| HMEC#1 | HMEC | HMEC | 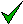 |
| MCF7_P | T est | T est | 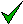 |
| MDA468 | T est | T est | 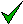 |
| MCF7_N | T est | T est | 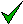 |
| HMEC#12 | HMEC | HMEC | 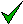 |
| MSSM_3 | MSSM | MSSM | 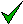 |
| MDA_231 | T est | T est | 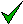 |
| MSSM_6 | MSSM | MSSM | 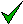 |
| MSSM_11 | MSSM | MSSM | 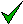 |
| MDA453 | T est | T est | 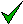 |

Accuracy:  100 %, (24/24)

Results of the classification of the MTSV1-7, MCF10A and MCF10F cell lines according to the model generated by HMECs, MSSMs and T-est cell lines.

| **Sample:** | **Prediction:** |
| --- | --- |
| MTSV1-7 | T est |
| MCF_10A | T est |
| MCF_10F | T est |
