# Supplementary material for: Transcription profiles of non-immortalized breast cancer cell lines
Source: BMC Cancer. 2006 Apr 20;6:99. doi: 10.1186/1471-2407-6-99 (PMC1524972; doi:10.1186/1471-2407-6-99)
Supplement: Additional File 6 — Table S3:.doc: SAM output. [file 1471-2407-6-99-S6.doc]

Table S3: SAM output

**Significant Genes List**

**Input Parameters**

Imputation Engine 5-Nearest Neighbor Imputer

Data Type Two Class, unpaired data

Data in log scale? FALSE

Number of Permutations 100

Blocked Permutation? FALSE

RNG Seed 1234567

(Delta, Fold Change) (1.52689, )

(Upper Cutoff, Lower Cutoff) (3.07394, -3.16356)

**Computed Quantities**

Computed Exchangeability Factor S0 0.001182868

S0 percentile 0

False Significant Number (Median, 90 percentile) (0.73718, 6.92949)

False Discovery Rate (Median, 90 percentile) (0.72988, 6.86088)

Pi0Hat 0.73718

**53 Positive Significant Genes**

|  |  |  |  |  |  |  |
| --- | --- | --- | --- | --- | --- | --- |
| **Gene Name** | **Gene ID** | **Score(d)** | **Numerator(r)** | **Denominator(s+s0)** | **Fold Change** | **q-value (%)** |
| IGFBP4 | M62403 | 9.787191 | 4320.843014 | 441.4793958 | 57.59544 | 0.72988068 |
| KRT19 | Y00503 | 9.311163 | 4897.571085 | 525.9891676 | 17.16797 | 0.72988068 |
| FES | X52192 | 8.917249 | 1031.794897 | 115.7077593 | 37.10035 | 0.72988068 |
| LAMA4 | X70904 | 7.826216 | 2757.074576 | 352.2870482 | 159.92483 | 0.72988068 |
| FCGRT | U12255 | 7.465785 | 2847.619205 | 381.4225917 | 6.21693 | 0.72988068 |
| FGFR1 | M37722 | 7.324378 | 1924.863795 | 262.8023674 | 1,925.86380 | 0.72988068 |
| MMP11 | X57766 | 6.745867 | 24783.51035 | 3673.880911 | 4.79001 | 0.72988068 |
| KRT18 | M26326 | 6.574484 | 5088.132141 | 773.9210988 | 3.36808 | 0.72988068 |
| TIMP1 | X03124 | 6.309959 | 25777.54513 | 4085.216011 | 4.22438 | 0.72988068 |
| PTPRS | U35234 | 6.218524 | 582.9347475 | 93.74165264 | 11.56885 | 0.72988068 |
| TIMP2 | J05593 | 6.136008 | 1471.722403 | 239.8501314 | 5.99639 | 0.72988068 |
| TGFBI | M77349 | 6.01303 | 5509.217141 | 916.213181 | 3.98677 | 0.72988068 |
| PLXNA3 | X87852 | 5.688799 | 1815.467837 | 319.1302382 | 2.07561 | 0.72988068 |
| ITGB3 | J02703 | 5.571132 | 1266.198667 | 227.2785129 | 71.86746 | 0.72988068 |
| KRT8 | M34225 | 5.426244 | 3508.715438 | 646.6194934 | 2.46063 | 0.72988068 |
| TCIRG1 | U45285 | 5.3497 | 992.4732108 | 185.5193982 | 15.08267 | 0.72988068 |
| TNFRSF1A | M33294 | 5.339195 | 1584.087117 | 296.6902273 | 2.96909 | 0.72988068 |
| MOX2 | X05323 | 5.174636 | 554.9981976 | 107.2535672 | 42.04464 | 0.72988068 |
| TP53I3 | AF010309 | 4.951985 | 1006.853145 | 203.3231343 | 6.53042 | 0.72988068 |
| BDNF | M61176 | 4.890176 | 597.0851825 | 122.098921 | 24.14945 | 0.72988068 |
| CDK5 | X66364 | 4.831046 | 842.4857256 | 174.3899169 | 1.52834 | 0.72988068 |
| CYR61 | AF031385 | 4.664608 | 1796.809291 | 385.2004948 | 3.52636 | 0.72988068 |
| VIM | X56134 | 4.518544 | 5761.317693 | 1275.038399 | 1.76881 | 0.72988068 |
| TYRO3 | D17517 | 4.366133 | 1208.12197 | 276.7029712 | 3.22907 | 0.72988068 |
| PSMB10 | X71874 | 4.216592 | 915.0193634 | 217.0044771 | 25.90521 | 0.72988068 |
| SPARC | J03040 | 4.122771 | 2176.639768 | 527.9555161 | 4.21054 | 0.72988068 |
| RGS4 | U27768 | 4.117293 | 625.5893064 | 151.9418958 | 7.68752 | 0.72988068 |
| GLIPR1 | X91911 | 3.993549 | 720.509564 | 180.4183663 | 3.59113 | 0.72988068 |
| HMGA1 | M23619 | 3.963658 | 3965.013757 | 1000.342043 | 1.75154 | 0.72988068 |
| COL8A1 | X57527 | 3.95484 | 718.5332675 | 181.6845197 | 24.85187 | 0.72988068 |
| NID | M30269 | 3.899057 | 591.7310751 | 151.7626019 | 6.26327 | 0.72988068 |
| GPR4 | U21051 | 3.891698 | 603.8536577 | 155.1645608 | 604.09978 | 0.72988068 |
| DAB2 | U53446 | 3.863494 | 1055.72461 | 273.2564484 | 8.90828 | 0.72988068 |
| COL6A1 | X15879 | 3.731967 | 2542.973504 | 681.4029145 | 4.57312 | 0.72988068 |
| MMP9 | J05070 | 3.731403 | 1372.941964 | 367.9426047 | 4.51114 | 0.72988068 |
| CDH2 | M34064 | 3.72673 | 909.0572917 | 243.9289333 | 908.92239 | 0.72988068 |
| CTGF | M92934 | 3.718956 | 853.7303362 | 229.5618162 | 19.64009 | 0.72988068 |
| NCOR2 | S83390 | 3.603011 | 1010.058873 | 280.3374675 | 1.93361 | 0.72988068 |
| GSTO1 | U90313 | 3.585805 | 4808.396824 | 1340.953214 | 1.69104 | 0.72988068 |
| ITGA5 | X06256 | 3.56462 | 3234.073982 | 907.2703551 | 3.49197 | 0.72988068 |
| AXL | M76125 | 3.536725 | 1530.568616 | 432.764381 | 2.25782 | 0.72988068 |
| IGFBP5 | M65062 | 3.491303 | 915.3422207 | 262.177851 | 27.63010 | 0.72988068 |
| SERPINB2 | M18082 | 3.388203 | 1481.630215 | 437.2908696 | 5.23167 | 0.72988068 |
| FOLH1 | M99487 | 3.382462 | 354.6508571 | 104.8499027 | 355.65086 | 0.72988068 |
| PRKCL1 | U33053 | 3.36791 | 667.7330341 | 198.263343 | 5.32072 | 0.72988068 |
| MGST2 | U77604 | 3.331793 | 452.2444103 | 135.7360467 | 9.01057 | 0.72988068 |
| DLG3 | U49089 | 3.299981 | 443.3390808 | 134.3459671 | 4.31120 | 0.72988068 |
| TNFRSF10D | AF029761 | 3.271556 | 1320.759943 | 403.7099792 | 6.15110 | 0.72988068 |
| FGF2 | M27968 | 3.225714 | 549.949035 | 170.4890994 | 8.74482 | 0.72988068 |
| MMP2 | J03210 | 3.223208 | 290.9295385 | 90.26087425 | 291.56633 | 0.72988068 |
| TXNRD1 | X91247 | 3.19368 | 878.9333147 | 275.2101906 | 6.57648 | 0.72988068 |
| ITGB8 | M73780 | 3.083795 | 1314.472894 | 426.2516745 | 1.97297 | 0.72988068 |
| VEGFC | U43142 | 3.073939 | 518.2080337 | 168.5811084 | 3.54873 | 0.72988068 |
|  |  |  |  |  |  |  |

**48 Negative Significant Genes**

|  |  |  |  |  |  |  |
| --- | --- | --- | --- | --- | --- | --- |
| **Gene Name** | **Gene ID** | **Score(d)** | **Numerator(r)** | **Denominator(s+s0)** | **Fold Change** | **q-value (%)** |
| SFN | AF029082 | -18.5106 | -12081.47014 | 652.6769468 | 0.00744 | 0.72988068 |
| SPINT2 | U78095 | -14.6598 | -4507.838042 | 307.4969038 | 0.02681 | 0.72988068 |
| KRT14 | J00124 | -12.0873 | -29250.46312 | 2419.926831 | 0.00390 | 0.72988068 |
| LAMB2 | S77512 | -11.3177 | -4423.179872 | 390.8209568 | 0.07984 | 0.72988068 |
| ITGA7 | X74295 | -11.0881 | -10321.96373 | 930.9065407 | 0.07677 | 0.72988068 |
| CDH3 | X63629 | -8.91054 | -3322.217843 | 372.8415478 | 0.08258 | 0.72988068 |
| IL1B | K02770 | -7.91974 | -3822.494195 | 482.654038 | 0.51679 | 0.72988068 |
| PLAU | M15476 | -7.91728 | -1910.496461 | 241.3072425 | 0.09647 | 0.72988068 |
| ITGB4 | X53587 | -7.91481 | -13820.65106 | 1746.176621 | 0.04527 | 0.72988068 |
| TPBG | Z29083 | -7.65776 | -1555.100973 | 203.0750894 | 0.18455 | 0.72988068 |
| RPS6KA1 | L07597 | -7.56221 | -2913.252594 | 385.2384168 | 0.29711 | 0.72988068 |
| KRT2A | M99061 | -7.43499 | -4996.876806 | 672.075974 | 0.21672 | 0.72988068 |
| EDG4 | AF011466 | -7.22249 | -996.1645489 | 137.925441 | 0.21312 | 0.72988068 |
| KRT16 | M21772 | -6.89211 | -920.5558696 | 133.5665389 | 0.01336 | 0.72988068 |
| IRF6 | AF027292 | -6.78544 | -1517.379209 | 223.6229207 | 0.00832 | 0.72988068 |
| LITAF | AF010312 | -6.72404 | -1922.701084 | 285.9442436 | 0.26788 | 0.72988068 |
| SEMA3F | U33920 | -6.45471 | -1424.612454 | 220.7090995 | 0.28155 | 0.72988068 |
| NOTCH1 | M73980 | -6.04979 | -1541.016531 | 254.722472 | 0.33860 | 0.72988068 |
| ITGA6 | X53586 | -5.93316 | -1046.521914 | 176.3851312 | 0.00095 | 0.72988068 |
| DSP | M77830 | -5.90152 | -1190.250451 | 201.6854559 | 0.00084 | 0.72988068 |
| CDA | L27943 | -5.43636 | -2996.035685 | 551.1109577 | 0.04756 | 0.72988068 |
| JUP | M23410 | -5.39362 | -5227.229237 | 969.1497271 | 0.14768 | 0.72988068 |
| JAG2 | AF003521 | -5.29494 | -1494.561654 | 282.2622237 | 0.23678 | 0.72988068 |
| KRT10 | M19156 | -4.99451 | -5275.46663 | 1056.252337 | 0.09690 | 0.72988068 |
| SERPINB1 | M93056 | -4.88524 | -1678.61433 | 343.6094345 | 0.16361 | 0.72988068 |
| BTG2 | U72649 | -4.86362 | -633.8764075 | 130.3301874 | 0.04109 | 0.72988068 |
| RARG | M24857 | -4.72989 | -843.5399407 | 178.342321 | 0.36925 | 0.72988068 |
| CDKN1A | U09579 | -4.62637 | -2813.185953 | 608.076604 | 0.60984 | 0.72988068 |
| BENE | U17077 | -4.5299 | -1977.063993 | 436.4473863 | 0.42570 | 0.72988068 |
| ERCC1 | M13194 | -4.40946 | -3119.72154 | 707.5059042 | 0.55758 | 0.72988068 |
| NGFR | M14764 | -4.1984 | -974.9751828 | 232.2253779 | 0.00103 | 0.72988068 |
| JUNB | M29039 | -4.06564 | -785.7228678 | 193.2592876 | 0.34277 | 0.72988068 |
| CAV1 | Z18951 | -3.9297 | -608.082607 | 154.7400773 | 0.37019 | 0.72988068 |
| GART | X54199 | -3.88729 | -836.0815258 | 215.0807957 | 0.29799 | 0.72988068 |
| CORO1A | D44497 | -3.87344 | -526.346941 | 135.8861133 | 0.16937 | 0.72988068 |
| IL1RN | M63099 | -3.78732 | -594.4865001 | 156.9677855 | 0.02053 | 0.72988068 |
| AREG | M30704 | -3.74395 | -388.6625243 | 103.8109489 | 0.10591 | 0.72988068 |
| CTSD | M11233 | -3.65793 | -2779.745129 | 759.9230897 | 0.41877 | 0.72988068 |
| DDB2 | U18300 | -3.63986 | -665.381175 | 182.804233 | 0.41074 | 0.72988068 |
| ITGA3 | M59911 | -3.61623 | -2882.162172 | 797.0078984 | 0.55400 | 0.72988068 |
| EGR1 | M62829 | -3.59122 | -2468.26395 | 687.3060643 | 0.46634 | 0.72988068 |
| FOS | K00650 | -3.52121 | -277.0842182 | 78.68997652 | 0.09651 | 0.72988068 |
| CAV2 | U32114 | -3.45734 | -933.7682138 | 270.0829137 | 0.46932 | 0.72988068 |
| STK24 | AF024636 | -3.35769 | -408.7948645 | 121.7488905 | 0.56449 | 0.72988068 |
| PDE4B | L12686 | -3.35594 | -300.5572055 | 89.55986597 | 0.00332 | 0.72988068 |
| KRT1 | M98776 | -3.25978 | -1963.121205 | 602.224156 | 0.09292 | 0.72988068 |
| EFNB2 | L38734 | -3.21521 | -281.4264308 | 87.52982517 | 0.04658 | 0.72988068 |
| FASN | S80437 | -3.16356 | -1363.204684 | 430.9080921 | 0.56565 | 0.72988068 |
