# Supplementary material for: Transcription profiles of non-immortalized breast cancer cell lines
Source: BMC Cancer. 2006 Apr 20;6:99. doi: 10.1186/1471-2407-6-99 (PMC1524972; doi:10.1186/1471-2407-6-99)
Supplement: Additional File 7 — Table S3:.doc: False discovery rates and p-values of the Pomelo Tool applied to the comparison HMEC-MSSM. [file 1471-2407-6-99-S7.doc]

Table S4: Pomelo Tool

Function call: /usr/local/Pomelo/multest Anova maxT 10000

Number of variables or genes: 75

Number of columns: 27

Type of test: Anova

MinP or MaxT?: maxT

Permutations used: 10000

Random seed: 1092253933

###############################################################

| Row ID unadj.p adj_p FDR_indep FDR_dep obs_stat abs(obs_stat) |
| --- |
| 5 FES 9.999e-05 0.0003 0.000312469 0.00153152 56.3971 56.3971 |
| 36 GPR4 9.999e-05 0.0003 0.000312469 0.00153152 38.0908 38.0908 |
| 74 SPARC 9.999e-05 0.0003 0.000312469 0.00153152 33.9865 33.9865 |
| 37 ITGB3 9.999e-05 0.0004 0.000312469 0.00153152 31.7551 31.7551 |
| 47 SPINT2 9.999e-05 0.0005 0.000312469 0.00153152 28.4054 28.4054 |
| 61 LAMA4 9.999e-05 0.0005 0.000312469 0.00153152 28.3269 28.3269 |
| 68 DAB2 9.999e-05 0.0005 0.000312469 0.00153152 27.5801 27.5801 |
| 51 TIMP1 9.999e-05 0.0005 0.000312469 0.00153152 26.2229 26.2229 |
| 12 SFN 9.999e-05 0.0007 0.000312469 0.00153152 22.7041 22.7041 |
| 75 TGFBI 9.999e-05 0.0007 0.000312469 0.00153152 22.3196 22.3196 |
| 34 CDH2 9.999e-05 0.001 0.000312469 0.00153152 21.5159 21.5159 |
| 29 ITGA6 9.999e-05 0.0015 0.000312469 0.00153152 20.6667 20.6667 |
| 35 DSP 9.999e-05 0.0028 0.000312469 0.00153152 19.0853 19.0853 |
| 17 PTPRS 9.999e-05 0.0028 0.000312469 0.00153152 19.0755 19.0755 |
| 14 IGFBP4 9.999e-05 0.0067 0.000312469 0.00153152 16.198 16.198 |
| 64 KRT14 9.999e-05 0.0085 0.000312469 0.00153152 15.3653 15.3653 |
| 32 FGFR1 9.999e-05 0.0085 0.000312469 0.00153152 15.3344 15.3344 |
| 66 KRT18 9.999e-05 0.0087 0.000312469 0.00153152 15.2361 15.2361 |
| 60 RGS4 9.999e-05 0.0092 0.000312469 0.00153152 14.9304 14.9304 |
| 67 KRT19 9.999e-05 0.0105 0.000312469 0.00153152 14.4647 14.4647 |
| 53 FGF2 0.00029997 0.0105 0.00068175 0.0033415 14.4555 14.4555 |
| 52 PSMB10 0.00029997 0.0116 0.00068175 0.0033415 13.9383 13.9383 |
| 38 MOX2 0.00029997 0.0123 0.00068175 0.0033415 13.5723 13.5723 |
| 24 IRF6 0.00019998 0.0123 0.0005555 0.0027227 13.5142 13.5142 |
| 69 NID 9.999e-05 0.0172 0.000312469 0.00153152 12.5785 12.5785 |
| 65 KRT16 9.999e-05 0.0175 0.000312469 0.00153152 12.5278 12.5278 |
| 30 ITGB4 0.00029997 0.0186 0.00068175 0.0033415 12.3155 12.3155 |
| 18 JUP 0.00019998 0.0193 0.0005555 0.0027227 12.2218 12.2218 |
| 42 CTSD 0.00019998 0.023 0.0005555 0.0027227 11.5757 11.5757 |
| 50 MMP9 9.999e-05 0.0296 0.000312469 0.00153152 10.7209 10.7209 |
| 7 TP53I3 0.00029997 0.0356 0.00068175 0.0033415 10.1803 10.1803 |
| 43 CTGF 0.00059994 0.0379 0.00124988 0.00612608 9.94093 9.94093 |
| 62 CDA 0.00029997 0.0435 0.00068175 0.0033415 9.61294 9.61294 |
| 8 BTG2 0.00069993 0.0445 0.00141878 0.00695393 9.5343 9.5343 |
| 48 GLIPR1 0.00079992 0.049 0.00157879 0.00773821 9.2758 9.2758 |
| 6 MMP11 9.999e-05 0.0616 0.000312469 0.00153152 8.71567 8.71567 |
| 22 TNFRSF10D 0.00109989 0.0641 0.00211517 0.0103672 8.58843 8.58843 |
| 57 COL8A1 0.00119988 0.0641 0.00224978 0.0110269 8.55292 8.55292 |
| 27 NGFR 0.00139986 0.0692 0.00249975 0.0122522 8.20753 8.20753 |
| 59 KRT8 0.00049995 0.0773 0.00110283 0.00540537 7.69953 7.69953 |
| 40 BDNF 0.00129987 0.079 0.00237781 0.0116545 7.63245 7.63245 |
| 19 RPS6KA1 0.00059994 0.0821 0.00124988 0.00612608 7.49657 7.49657 |
| 28 ITGA5 0.0069993 0.0854 0.0100951 0.0494799 7.33645 7.33645 |
| 26 PLXNA3 0.00169983 0.0854 0.00296482 0.0145316 7.30233 7.30233 |
| 15 IGFBP5 0.0029997 0.0854 0.00511313 0.0250612 7.19172 7.19172 |
| 11 CAV2 0.00519948 0.0854 0.00829704 0.0406668 7.10472 7.10472 |
| 33 EDG4 0.00419958 0.0866 0.00684714 0.0335603 6.89939 6.89939 |
| 31 ITGA7 0.0059994 0.0941 0.00911674 0.0446844 6.60965 6.60965 |
| 73 BENE 0.00619938 0.1063 0.00911674 0.0446844 6.34566 6.34566 |
| 54 TIMP2 0.00379962 0.1088 0.0063327 0.0310388 6.17752 6.17752 |
| 16 PRKCL1 0.00619938 0.122 0.00911674 0.0446844 5.87539 5.87539 |
| 56 PLAU 0.00589941 0.1327 0.00911674 0.0446844 5.60728 5.60728 |
| 55 COL6A1 0.0112989 0.1442 0.014363 0.070398 5.38063 5.38063 |
| 63 LAMB2 0.00989901 0.1574 0.013025 0.0638402 5.08197 5.08197 |
| 10 TCIRG1 0.00989901 0.1574 0.013025 0.0638402 5.05353 5.05353 |
| 45 VEGFC 0.00929907 0.1574 0.0129154 0.0633028 4.97198 4.97198 |
| 25 CDH3 0.0111989 0.1631 0.014363 0.070398 4.77277 4.77277 |
| 3 TYRO3 0.0122988 0.18 0.0153735 0.0753508 4.52879 4.52879 |
| 20 TNFRSF1A 0.00929907 0.18 0.0129154 0.0633028 4.52692 4.52692 |
| 58 KRT10 0.0139986 0.18 0.0172114 0.0843592 4.40709 4.40709 |
| 13 CAV1 0.0187981 0.1895 0.0216901 0.106311 4.25402 4.25402 |
| 2 TPBG 0.0175982 0.1895 0.0206229 0.10108 4.1978 4.1978 |
| 71 TXNRD1 0.0173983 0.1895 0.0206229 0.10108 4.09519 4.09519 |
| 9 LITAF 0.00979902 0.1895 0.013025 0.0638402 3.97127 3.97127 |
| 44 FCGRT 0.0288971 0.1895 0.0318718 0.156215 3.92514 3.92514 |
| 49 JAG2 0.020098 0.1895 0.0228386 0.11194 3.85812 3.85812 |
| 70 KRT1 0.0249975 0.1895 0.0279823 0.137151 3.69979 3.69979 |
| 46 SERPINB1 0.029697 0.1895 0.0322794 0.158213 3.42283 3.42283 |
| 23 EGR1 0.0355964 0.1895 0.038139 0.186933 3.38991 3.38991 |
| 1 JUNB 0.0393961 0.1895 0.0410376 0.20114 3.27849 3.27849 |
| 41 CYR61 0.0386961 0.1895 0.0408762 0.200349 3.17391 3.17391 |
| 4 NOTCH1 0.0147985 0.1895 0.0179014 0.0877413 3.10849 3.10849 |
| 21 RARG 0.0863914 0.2136 0.0887582 0.435036 2.24359 2.24359 |
| 72 KRT2A 0.141486 0.2478 0.143398 0.702844 1.84097 1.84097 |
| 39 SEMA3F 0.19578 0.2478 0.19578 0.959589 1.63816 1.63816 |
